# Hidden charisma: ethno-ornithological knowledge and attitudes of local communities toward a threatened endemic pheasant in the Upper Blue Nile Basin, Ethiopia

**DOI:** 10.1186/s13002-026-00942-0

**Published:** 2026-09-17

**Authors:** Abadi Mehari Abrha, Kai Gedeon, Lars Podsiadlowski, Temesgen Tigab Derso, Tesfaye Mesele, Till Töpfer

**Affiliations:** 1 Institute for Evolutionary Biology and Ecology, University of Bonn, An der Immenburg 1, 53121 Bonn, Germany; 2 Museum Koenig Bonn, Leibniz Institute for the Analysis of Biodiversity Change, Adenauerallee 127, 53113 Bonn, Germany; 3 Department of Biodiversity and Ecotourism Management, Mekelle University, P.O. Box 231, Mekelle, Tigray, Ethiopia; 4 Ethiopian Biodiversity Institute, P.O. Box 30726, Addis Ababa, Ethiopia; 5 Department of Foreign Languages and Literature, Mekelle University, P.O. Box 231, Mekelle, Tigray, Ethiopia

**Keywords:** Attitude, Conservation, Ethno-ornithology, Charisma, Harwood’s Francolin, traditional ecological knowledge (TEK)

## Abstract

**Background:**

Ethno-ornithological knowledge plays a key role in the conservation and management of birds and their habitats. However, the cultural roles and significance of birds in Ethiopia, as well as the threats to their survival within human cultural contexts remain largely underexplored. This study investigated the socio-economic, demographic, spatiotemporal and behavioral factors that influence the knowledge and attitude of local communities toward Harwood’s Francolins in the Upper Blue Nile Basin.

**Methods:**

We conducted a quantitative survey with questionnaires administered to 379 households, along with qualitative data collected in 2019–2020. We used logistic regression models in SPSS and thematic analysis to examine the determinants of respondents’ knowledge and attitudes toward the species and triangulate the data by cross-referencing both methods.

**Results:**

This study found that respondents’ knowledge of and attitudes toward the status and value of Harwood’s Francolin were influenced by marital status, distance and existing relationship (*p* < 0.05). Gender and length of residency (*p* < 0.05) were key factors influencing respondents’ knowledge of hunting pressures on the species. Education level was the only factor influencing knowledge of indirect disturbances (*p* < 0.05). Given these threats, most respondents strongly supported the proposed conservation measures, with women showing higher support (*p* < 0.05). Education, livelihood and distance also significantly affected knowledge of conservation action (*p* < 0.05). This research shows that the bird’s role in Ethiopian society extends beyond biology to symbolic, medicinal, and ritual significance.

**Conclusion:**

The local communities demonstrated extensive knowledge of and attitudes toward the status and value on Harwood’s Francolin. The charismatic nature of the species, combined with its ecological and cultural relevance, fosters strong local support for its conservation, illustrating how closely biodiversity and human culture are linked. In addition, the local communities also acknowledged that the species has been declining and even extirpated in some previously known sites due to several anthropogenic disturbances. Consequently, conservation efforts should prioritize raising awareness among local communities about the restrictions on hunting and egg collection during breeding seasons, while also generating alternative income through the establishment of protected areas. In addition, we underscore the importance of gender-inclusive conservation strategies and recommend for comprehensive research on Harwood’s Francolin and related pheasant species, as well as other wildlife, within the context of Ethiopia’s cultural and social systems.

**Supplementary Information:**

The online version contains supplementary material available at https://doi.org/10.1186/s13002-026-00942-0.

## Background

Ethno-ornithology encompasses the diverse relationships between humans and bird species within ecosystems, highlighting the indigenous knowledge and attitudes of local communities toward birds in relation to culture and spirituality [1, 2]. These factors also play a crucial role in research and raise awareness for the conservation and management of bird species [2]. Ethno-ornithological relationship studies have centered into five main categories: (i) aesthetic and cultural values [3, 4]; (ii) ethno-medicine [5–8]; (iii) food or bushmeat [6, 9–11]; (iv) weather forecasting [12–14]; and (v) human-bird conflict [15–18]. All these relationships (or interactions) are manifestations of human culture, reflecting the deep connections between birds and society [1, 2].

Perceptions of birds, whether positive, neutral, or negative, are linked to their ecological roles and interactions with human communities. The awareness and perception of threatened bird species by local communities are essential for developing coexistence strategies [19], community-based conservation [20, 21], management [20] and education [18, 21–22]. Conversely, the lack of knowledge and negative attitudes of people toward birds, a phenomenon often framed as human–bird conflict, can threaten both the birds and the broader ecosystem [2, 17] and this has also prompted several studies aimed at supporting conservation efforts [16–18].

Unlike birds of prey, which are often negatively perceived by many societies [e.g., 18], galliform species are often valued positively in human culture, primarily for their value as a food source. This interaction has caused the global decline of many galliform species [8, 9], such as partridges [21, 23], grouses [21], and francolins [24]. This phenomenon is also observed in our study species, Harwood’s Francolin (*Pternistis harwoodi* (Blundell & Lovat, 1899)), with local communities engaging in subsistence hunting using traditional traps known locally as ‘Wesfentir’, ‘Diftosh’ and ‘Ashkila’, and degrading its habitat through agricultural expansion and resource extraction [25–27]. This exacerbates the challenges for endemic bird species that have a specialized diet and limited dispersal ability [28–30], such as Harwood’s Francolin. Consequently, Harwood’s Francolin is currently classified as Near Threatened throughout its range globally, with a continuing population decline that raises serious conservation concerns [31].

Due to their large body size, ground-dwelling habits, cultural and subsistence importance, and frequent proximity to human settlements, Galliformes have been among the most heavily hunted bird groups since antiquity to contemporary times [32–34]. Consequently, hunting, combined with habitat loss, has driven significant declines in population size and thus increased the extinction risk of many species globally [32], including Harwood’s Francolin [25, 26, 35].

Despite long-standing interactions between humans and birds [1], ethno-ornithological knowledge in bioculturally heterogeneous countries such as Ethiopia remains poorly documented. This gap limits integration with ecological evidence, constraining the development of robust, evidence-based, and culturally informed conservation strategies for pheasants, including Harwood’s Francolin. Understanding how local cultural practices influence bird populations is therefore essential, particularly for galliform species [e.g., 8, 21], yet systematic research in Ethiopia remains scarce. Harwood’s Francolin holds cultural and social significance in Ethiopia. Despite this, only biological and ecological evidence of the species have been documented over the last three decades [25, 27, 35]. Specifically, the species is a ground-foraging omnivore in the Upper Blue Nile Basin. By consuming seeds, invertebrates, fruits, rhizomes, and underground plant parts [25; 36], it likely contributes to seed dispersal and seed predation, while its scratching and foraging activities promote soil turnover and nutrient cycling, as is typical of ground-dwelling galliform birds. Furthermore, it forms an integral part of the food web by serving as prey for aerial and terrestrial predators, including humans [36]. However, its wider ecosystem functions have not been systematically quantified and are instead inferred from descriptive and field observations [25, 36].

Unlike the plethora of evidence from ethnobotanical [37–39] and some ethnozoological [40] community level studies, this paper presents the first and detailed species-level ethno-ornithological research in Ethiopia and advances understanding of bird–human cultural interactions, aiming to provide more comprehensive support for future conservation initiatives. However, approximately 6–20 bird species are estimated to be traded for traditional medicinal purposes in Ethiopia, although the estimate is based on coarse continental assessments rather than detailed national studies [8].

In this paper, we therefore, examined the knowledge and attitudes of local communities regarding status and value of the species, disturbance type, and conservation action, drawing on the interactions described above. Our objective was to investigate the socio-economic, demographic, spatiotemporal, and behavioral factors that influence the knowledge and attitude of local communities toward Harwood’s Francolins in the Upper Blue Nile Basin (Table 1). This study tested four hypotheses. First, we hypothesized that socio-economic and demographic characteristics of respondents such as age, gender, education, marital status, and household size influence their knowledge of and attitudes toward the status, value, threat, and conservation action of the species (H1). Second, local communities living in and adjoining to the habitat of Harwood’s Francolins have greater knowledge of and more favorable attitudes toward the species than those living farther away (H2). Third, the existing relationship with game birds significantly influences both ethno-ornithological knowledge of and attitudes toward the target species and related huntable pheasants (H3). Last, local communities inhabit for longer residency period in the area have greater knowledge about the target species compared to residents that are more recent (H4).

## Methods

### Study area

**Fig. 1:**
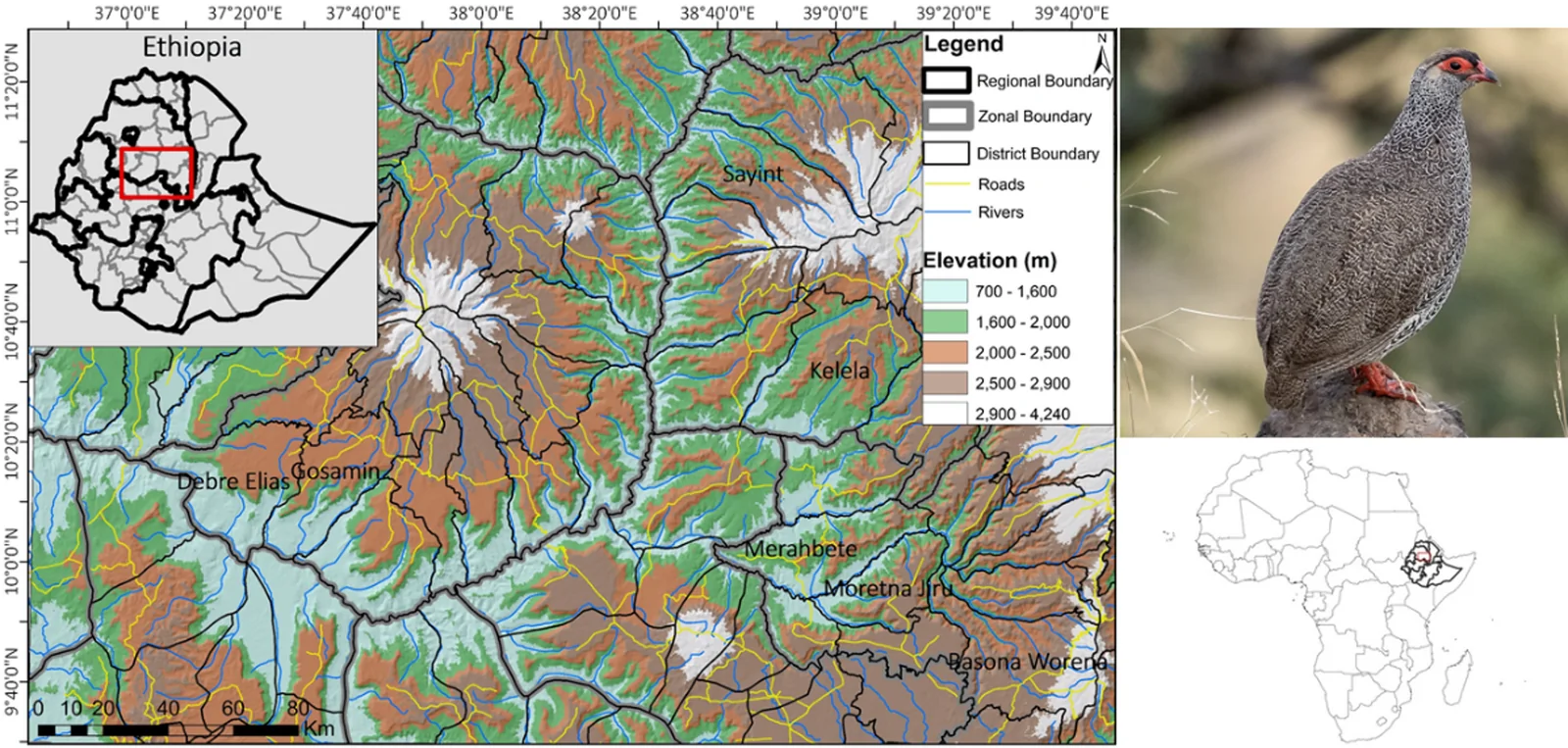
Map of the study area and picture of Harwood’s Francolin (© Mathieu Bally, https://ebird.org/species/harfra4)

The study area lies within 9°40′0″–11°20′0″N and 37°0′0″–39°40′0″E in the Upper Blue Nile Basin (UBNB). The elevation ranges from 700 to 4240 m a.s.l., and the terrain is highly variable, with steep, mountainous, rocky, and rugged features (Fig. 1). The area can be described as human-dominated landscape in dry evergreen montane forest and evergreen scrub ecosystems and has distinct scrublands and acacia woodlands. The UBNB is part of the Sudan–Guinea Savanna and Afrotropical Highlands biomes [41]. Due to its high avian diversity and the presence of rare species, including Harwood’s Francolin, four Important Bird Areas (IBAs) have been designated within the basin: Jemma and Jara Valleys, Mid-Abbay (Blue Nile) River Basin, Choke Mountains, and the Guassa Community Conservation Area [41]. The most recent total human population of the study areas was 836,918; of this population, 81.3% were followers of Orthodox Christianity, 94.4% lived in rural areas, and 95.7% belonged to conventional households [42]. Thus, demographic characteristics including religion, culture and language were excluded from this study, as the population was largely homogeneous with respect to these characteristics.

### Sampling design and data collection

The survey was conducted in seven districts of the UBNB in the end of 2019 and beginning of 2020. Earlier field expeditions (2015–2018) associated to this survey also helped us reshape and design the questionnaires in many areas of the basin. While Harwood’s Francolins were observed in multiple locations throughout the basin, questionnaire distribution was limited to representative districts due to the simultaneous conduct of other biological and ecological research activities [27, 43]. In each district, we selected specific villages randomly to collect data on knowledge and attitude of local people toward the species in the areas. The sample areas were Gosamin, Debre Elias, Merahbete, Moretna Jiru, Basona Worena, Kelela, and Sayint districts. Prior to questionnaire distribution, discussions were held between the researchers and community leaders (village chairpersons) regarding the aims of the study. Both discussants were agreed about the study and accepted to inform the members of the household in the selected areas. Subsequently, a pilot survey involving 20 respondents was conducted to verify the appropriateness and conciseness of the questionnaire for the target stakeholders and most questions related to this survey were also tested in 2016 in Merahbete district.

We used systematic random sampling technique [44] to select respondents from both existed lists provided by the leaders and compiled lists by researchers. A closed-ended structured questionnaire was administered, and the questions were translated into Amharic to make them easily understandable to respondents. The sample size for the total population of 836,918, as reported by the Central Statistical Authority [42], was calculated using Eq. 1 [45], and a finite population correction (FPC) [46, 47] was not applied because the population was assumed to be infinite.

  

1 $$\:{n}_{0}=\frac{{Z}^{2}p\:\left(1-p\right)}{{e}^{2}}$$

Where $\:{n}_{0}$ is the sample size, $\:{Z}^{2}$ is the square of the z-score corresponding to the desired confidence level (for example, for a 95% confidence level, Z = 1.96, and $\:{Z}^{2}$ = 3.8416), *e* is the desired margin of error, and *p* is the estimated population proportion. We chose *p* = 0.5 because it maximizes the variability$\:\:1-p$, resulting in the largest sample size and ensuring a reliable margin of error.

Thus, the minimum required sample size was 384 respondents. To account for possible non-response, structured questionnaires were administered to 400 respondents initially, of which 379 were fully completed and returned. Some questionnaires (5.3%) that were partially completed or not returned after distribution were excluded from further analysis (Supplementary Table S1).

### Items and scoring system of the questionnaires

At the beginning of the questionnaire, nine explanatory variables were included, covering the respondents’ socio-economic, demographic, spatiotemporal (distance and residency period), and behavioral (prior experience) variables (Table 1). Next to this section, we constructed 29 items (questions) belonging to five categories (i.e., response variables) related to the knowledge and attitudes of local communities toward the species (Supplementary Table S2). These included:

1. Status of the species.
2. Value of the species.
3. Hunting pressure.
4. Habitat destruction.
5. Conservation action.

For responses, we used two non-comparative scaling techniques, viz., simple category and itemized rating scales. The former scale (Category 1) consisted of items with trichotomous options (yes = 1, no = 2, and unsure = 3) that focused on the status of the species. This category included three items related to respondents’ knowledge of species occurrence, endemic status, and population trends. The latter (Categories 2–5) incorporated Likert-scale items [48–50], assessing respondents' knowledge and attitudes toward Harwood’s Francolins regarding the value of the species (seven items), hunting pressure (two items), habitat destruction (11 items), and conservation action (six items) (Supplementary Table S2). To better understand human pressures affecting the species and its habitat, disturbance types were classified as direct disturbance (hunting and egg collecting) and indirect disturbance (habitat destruction). We defined habitat destruction as the loss of habitat components due to agricultural operations, settlement, mining activities, firewood collection, and associated threats, as clearly documented in the literature [e.g., 51]. Therefore, most variables (Categories 2–5) were gauged by Likert’s measurement model, with a scoring system divided into symmetric (neural or undecided) versus asymmetric (skewed) dimensions [50]. Thus, our measurement scale incorporated sequential statements with five psychometric alternatives: strongly disagree = 1, disagree = 2, undecided = 3, agree = 4, and strongly agree = 5 [49, 50, 52, 53].

Alongside the quantitative questionnaire survey, qualitative data were collected through Focus Group Discussions (FGDs) and Key Informant Interviews (KIIs) involving 15 and 10 participants, respectively, focusing on selected key items from the category “hunting pressure” and “value of the species”, variables wellknown across the study area. The participants were chosen based on long-term residency, prior experience, and knowledge of the species, with 1–3 participants selected from each district. These qualitative methods were employed to contextualize of the survey findings and to achieve methodological triangulation [sensu 54], thereby enhancing the credibility and depth of the study. A non-probability sampling technique [44] was used to select herders, farmers, clergymen, and scholars to explore the medicinal value of the species (through KIIs) and its mythological value (through FGDs).

### Data analyses

The questionnaire data was transcribed from Amharic to English and the data were coded and checked for correctness in Microsoft Office Excel 2016 spreadsheet and exported to SPSS (version 20; IBM SPSS Inc, USA) to undertake analyses. Initially, our data were recoded for ease of interpretability and to test the predictive performance of models. To improve statistical robustness and model efficiency while enhancing interpretability, all continuous variables in Table 1 were not grouped. The categorical variables were recoded and dichotomized as follows: (1) marital status, married (0) and unmarried (1); (2) education level, non-literate (0) and literate (1); (3) major livelihood, resource-based livelihoods (agricultural activities and natural resource extraction) (0) and non-resource-based livelihoods (employed, business, and others) (1). Gender and existing relationship (i.e., prior experience) with species were retained in their original codes. All categorical variables were treated as dummy variables in the regression analyses.

We used descriptive statistics to summarize socio-economic and demographic characteristics of the respondents, as well as their responses regarding their knowledge of and attitudes toward the species. Knowledge items assessing the occurrence of the species, endemic status, and population trend, with trichotomous response options, were analyzed using frequencies and percentages to evaluate respondents’ level of awareness. Similarly, other items measured on a five-point Likert scale were combined into three ordinal scales: disagree, neutral, and agree, to assess overall trends in knowledge and attitudes of respondents. For logistic regression analyses, the response variables including status of the species recorded as a trichotomous data was combined into binary categories (yes = 1 and no and unsure = 0). We retained the five-point Likert scale for all other variables (i.e., value of the species, hunting pressure, habitat destruction, and conservation action) in the analyses. The internal consistency of the items was assessed using Cronbach’s alpha reliability coefficient [55] to evaluate scale reliability and unidimensionality, thereby informing subsequent analyses [56]. The alpha ranges from 0 to 1 with acceptable values lies between 0.7 and 0.95 [e.g., 57]. The alpha value below the acceptable level mainly near to 0 implies there is little self-consistency and association between items. However, alpha value close to 1 may indicate redundancies and does not necessarily verify high internal consistency between items [58]. The inter-item correlation matrix and Cronbach’s Alpha if item deleted were checked to determine whether the reliability of the questionnaire could increase or decrease. Thus, we retained items to avoid exaggeration and redundancy [56, 58]. Accordingly, the Cronbach’s alpha coefficients for all response variables were between 0.74 and 0.93 (Supplementary Table S2), suggesting evidence of reliability for all items of each variable. We also used Spearman’s rank correlation coefficients(r_s_) to determine the relationship among variables.

We applied a log_10_ transformation to continuous variables and tested for multicollinearity among explanatory variables using variance inflation factors (VIF values < 2). Binary logistic regression was used to examine the relationship between a binary response variable and the explanatory variables, and model fit was assessed using the Hosmer–Lemeshow goodness-of-fit test. After verifying model assumptions, including the test of parallel lines, we applied ordinal logistic regression with a cumulative logit link function to analyze the ordinal data and identify key determinants, such as gender, age, marital status, household size, education level, major livelihood, distance to habitats, residency period, and existing relationship regarding knowledge of and attitudes toward Harwood’s Francolins. Thematic analysis was used to triangulate the data by comparing and cross-referencing findings from both the logistic regression models and qualitative themes. This allowed us to validate and deepen our understanding of the factors influencing knowledge and attitudes, ensuring the reliability and accuracy of our results. Statistical significance was determined using a two-tailed test with a significance level of α = 0.05.

**Table 1:** Variables used to analyze the knowledge and attitudes of the local people toward Harwood’s Francolins

Variable	Type of variable	Category of variable	Details	Relationship	Variable codes and groups^a^	Respondents (*N*)	Percent
Gender	Categorical	Demographic	Sex of respondents	Positive/Negative	Female = 0	118	31.1
					Male = 1	261	68.9
Age	Continuous	Demographic	Ages of respondents per years	Positive	18–30	124	32.7
					31–55	223	58.8
					56+	32	8.4
Marital status	Categorical	Demographic	The relationship of respondents	Positive/Negative	Married = 0	279	73.6
					Single = 1	50	13.2
					Divorced = 2	24	6.3
					Widowed = 3	14	3.7
					Widower = 4	12	3.2
Household size	Continuous	Demographic	The number of individuals in the family	Positive	1–3	155	40.9
					4–7	174	45.9
					8+	50	13.2
Education level	Categorical	Socio-economic	The current educational attainment of respondents	Positive	Non-literate = 0	216	57.0
					Primary school = 1	91	24.0
					Secondary school = 2	44	11.6
					First degree = 3	21	5.5
					Second degree and above = 4	7	1.9
Major livelihood	Categorical	Socio-economic	The key livelihood of respondents	Negative	Resource based activities = 0 (agriculture and natural extraction)	290	76.5
					Employed = 1	45	11.9
					Business = 2	24	6.3
					Others = 3	20	5.3
Distance to habitat (km)	Continuous	Spatial	The estimated distance between the settlement and habitat of the species	Negative	0-2.5	250	66.0
					2.6-5.0	89	23.5
					5.1+	40	10.5
Residency period (yr)	Continuous	Temporal	The total number of years lived by respondents	Positive	1-10.9	118	31.1
					11-20.9	39	10.3
					21+	222	58.6
Existing relationship	Categorical	Behavioral	The prior experience in terms hunting, egg, collecting, symbolic and cultural aspects	Positive	Yes = 0	362	95.5
					No = 1	17	4.5

^a^variables were grouped to show comparisons with levels of participation in the questionnaire survey

## Results

### Characteristics of respondents

The detailed socio-economic, demographic, spatial and behavioral variables related to the respondents for this study are given in Table 1. In our study, 400 questionnaires were distributed of which 379 questionnaires were completed and included in the analysis, whereas 21 were excluded. In this survey, 68.9% of respondents were males and most respondents were aged 31–55 (58.8%), with an overall mean age of 38.15 ± 11.95 years (mean ± SD). The majority of respondents were married (73.6%). The household size of the respondents was ranged from 1 to 12 members, with nearly half (45.9%) having 4–7 members and their educational background indicated that 57.0% were non-literate. The livelihood of most respondents was resource-based (76.5%), including agriculture and forest-based subsistence activities. The average residency period and distance between the respondents and habitat of the species was 25.4 ± 17.1 years and 2.3 ± 2.0 km (mean ± SD), respectively. Nearly all respondents had an existing relationship with the study species (95.5%).

### Status of Harwood’s Francolins

Most respondents were aware of the presence of the species (76.5%; *n* = 290), endemic status (70.4%; *n* = 267) and population trend (65.2%; *n* = 247) in the Upper Blue Nile Basin in Ethiopia (Supplementary Table S2). The status of the species was negatively associated with marital status (*r* = − 0.10; *p* < 0.05), while it was positively correlated with distance (*r* = 0.35; *p* < 0.001) and prior experience with the species and other game bird species in the area (*r* = 0.51; *p* < 0.001). The binary logistic regression model showed adequate model fit (Hosmer–Lemeshow goodness-of-fit test = 12.13, df = 8, *p* > 0.05). Marital status had significant effect on the knowledge of respondents of toward the status of the species (β = -0.744; *p* < 0.05). Married respondents were less likely to have the knowledge of the species compared to unmarried respondents. Distance was also a significant variable for the status of the species. The proximity of respondents to the habitat of species (β = -0.845, *p* < 0.05) was associated with higher knowledge about the status of the species and respondents living farther away had lower odds of having knowledge, with each unit increase in distance corresponding to a 57% decrease in the odds (OR = 0.429, 95% CI: 0.198–0.931). Similarly, having a relationship with the species was a determinant variable (β = 2.695, *p* < 0.001). Respondents with prior experience were about 14.8 times more likely to have the knowledge of occurrence, endemic status and population trends compared to those without such a prior connection (OR = 14.806, 95% CI: 6.299–34.798) (Table 2).

**Table 2:** Binary logistic regression results for variables affecting respondents’ knowledge of Harwood’s Francolins in the Upper Blue Nile Basin. The reference categories used for comparison in the model were gender (male = 1), marital status (unmarried = 1), education level (literate = 1), major livelihood (non-resource-based livelihoods = 1), and existing relationship (no = 1)

Gender	β	SE	Wald	Odds ratio (95% CI)	*p*-value
	0.008	0.303	0.001	1.008 (0.557, 1.826)	0.978
Age	-1.517	1.216	1.559	0.219 (0.020, 2.378)	0.212
Marital status	-0.744	0.337	4.869	0.475 (0.245, 0.920)	0.027*
Household size	0.541	0.598	0.819	1.718 (0.532, 5.546)	0.365
Education level	0.149	0.387	0.149	1.161 (0.544, 2.478)	0.700
Major livelihood	0.404	0.365	1.226	1.498 (0.732, 3.063)	0.268
Distance	-0.845	0.395	4.587	0.430 (0.198, 0.932)	0.032*
Residency period	0.284	0.380	0.558	1.328 (0.631, 2.798)	0.455
Relationship	2.695	0.436	38.240	14.806 (6.299, 34.798)	< 0.001***

Significance: * < 0.05; ** < 0.01; ***p < 0.001

### Value of the target species

**Fig. 2:**
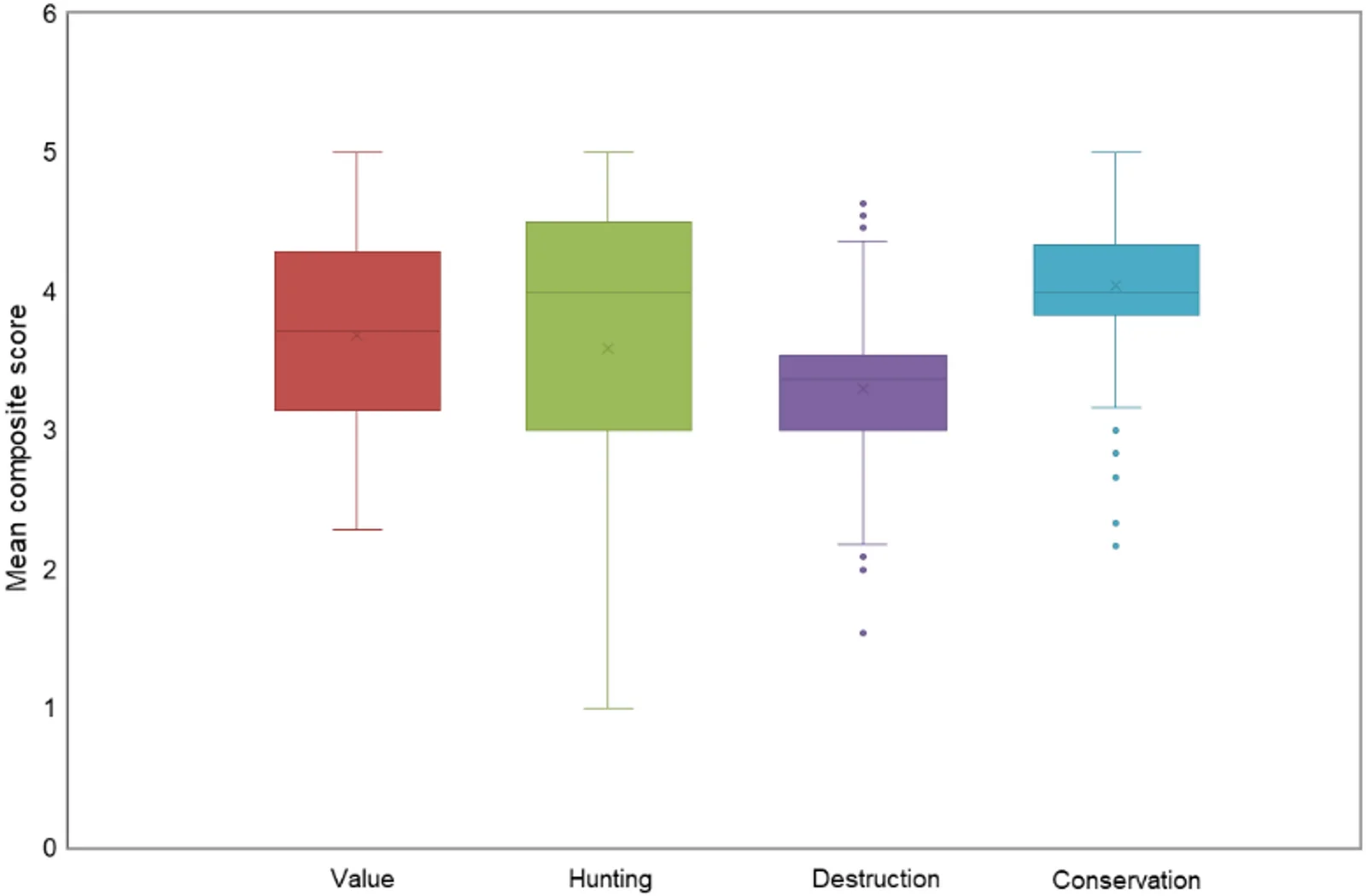
Mean composite scores of knowledge and attitudes of local communities toward the species based on four dependent variables, measured using Likert-scale responses (*n* = 379)

Based on the composite score, most respondents agreed on the value of the species (median = 3.7; interquartile range = 3.1–4.3) (Fig. 2). When analyzed item by item, the top-rated items were as follows: 93.4% of respondents (*n* = 354) rated the species as important for household subsistence; 64.4% (*n* = 244) recognized its trademark, aesthetic, and symbolic significance; and 59.9% (*n* = 227) valued it for market exchange (Supplementary Table S2). The value of the species was positively associated with marital status (*r* = 0.11; *p* < 0.05), while it was negatively significantly correlated with distance (*r* = -0.27, *p* < 0.001) and relationship (*r* = -0.28; *p* < 0.001). The test of parallel lines assumption showed that the ordinal logistic regression model investigating the value of the species was satisfied (χ² = 22.31, df = 18, *p* > 0.05). Marital status significantly influenced perceptions of the species, with married participants (β = -0.633, *p* < 0.05, OR = 0.463) more likely to hold negative attitudes toward it. Distance was also a significant predictor (β = -0.861, *p* < 0.01, OR = 0.423), with those living in close proximity to the habitat of the species having more positive attitudes toward its value. Prior experience significantly influenced respondents’ attitudes toward the value of the species (β = 1.054, *p* < 0.01, OR = 2.869), with respondents who had prior experience being 2.87 times more likely to express a positive attitude than those without (Table 3).

**Table 3:** Ordinal logistic regression results for determinants of the value of the species and hunting pressure

Variable	β	SE	Wald	Odds ratio (95% CI)	*p*-value
Value					
Gender	-0.129	0.235	0.301	0.879 (0.555, 1.393)	0.583
Age	-0.769	0.933	0.680	0.463 (0.074, 2.885)	0.410
Marital status	-0.633	0.248	6.527	0.531 (0.327, 0.863)	0.011*
Household size	0.278	0.475	0.341	1.320 (0.520, 3.350)	0.559
Education level	0.297	0.310	0.917	1.346 (0.733, 2.471)	0.338
Livelihood	-0.003	0.298	0.000	0.997 (0.556, 1.788)	0.992
Distance	-0.861	0.311	7.646	0.423 (0.230, 0.778)	0.006^**^
Residency period	0.179	0.319	0.314	1.196 (0.640, 2.235)	0.575
Relationship	1.054	0.338	9.748	2.869 (1.479, 5.565)	0.002^**^
Hunting pressure					
Gender	-1.141	0.222	26.307	0.319 (0.207, 0.494)	< 0.001***
Age	0.065	0.860	0.006	1.067 (0.198, 5.758)	0.940
Marital status	-0.192	0.226	0.720	0.825 (0.530, 1.285)	0.396
Household size	0.338	0.439	0.591	1.402 (0.593, 3.315)	0.442
Education level	-0.247	0.285	0.751	0.781 (0.447, 1.366)	0.386
Livelihood	-0.239	0.275	0.759	0.787 (0.459, 1.350)	0.384
Distance	-0.026	0.286	0.008	0.974 (0.556, 1.707)	0.928
Residency period	1.175	0.301	15.241	3.238 (1.795, 5.841)	< 0.001***
Relationship	0.429	0.298	2.069	1.536 (0.856, 2.754)	0.150

Significance: * < 0.05; ** < 0.01; ***p < 0.001

### Hunting pressure

In general, the majority of respondents agreed on the impact of hunting pressure on the species (median = 4; interquartile range = 3.0-4.5) (Fig. 2). When examined by item, most respondents were aware that hunting (64.4%, *n* = 244) is one of the factors contributing to the decline of species in their natural habitats. Additionally, nearly half of respondents (47.0%, *n* = 178) indicated that egg collecting also affects the species (Supplementary Table S2). Knowledge of direct disturbances mainly hunting and egg collecting was positively correlated with gender (*r* = 0.30; *p* < 0.001) and length of residency (*r* = 0.26; *p* < 0.001). Model assumption for the ordinal logistic regression of hunting pressure showed model fit (χ² = 39.57, df = 27, *p* > 0.05). Gender significantly influenced knowledge of hunting pressure and egg collecting, with women less likely to know that these factors decrease Harwood’s Francolins. The logistic regression also showed that residency period (β = 1.175, OR = 3.238, *p* < 0.001) was the most determinant predictor for the knowledge of the local communities in relation hunting practices (Table 3).

### Habitat destruction

Most respondents showed a neutral response to the type of habitat destruction hypothesized to influence the species indirectly (median = 3.4; interquartile range = 3.0-3.5) (Fig. 2). Item-wise analysis revealed that the top-ranked items linked to habitat destruction, according to the respondents, were cutting trees for firewood (86.5%, *n* = 328), denudation of habitats by farming activities (69.7%, *n* = 264), vegetation clearance for fencing and house construction (61.7%, *n* = 234), and charcoal preparation mainly for market exchange (56.7%, n = 215). Nearly half of respondents also identified livestock grazing as an important factor (48.8%, *n* = 185) (Supplementary Table S2). Spearman’s correlation analysis showed that only habitat destruction was positively correlated with education level (*r* = 0.12; *p* < 0.05). The test of parallel lines for the ordinal logistic regression of habitat destruction demonstrated adequate fit (χ² = 14.94, df = 18, *p* > 0.05). The results showed that only education level was a determinant variable for the habitat destruction (β = -0.692, OR = 0.501, *p* < 0.05) (Table 4). Specifically, educated respondents showed greater knowledge of the factors influencing the species in the context of habitat destruction (Table 4).

**Table 4:** Explanatory variables significantly affecting knowledge and attitudes of local communities toward habitat destruction and conservation action

Variable	β	SE	Wald	Odds ratio (95% CI)	*p*-value
Habitat destruction					
Gender	0.127	0.238	0.283	1.135 (0.712, 1.810)	0.595
Age	0.875	0.953	0.843	2.399 (0.371, 15.532)	0.359
Marital status	0.203	0.250	0.655	1.225 (0.751, 2.000)	0.418
Household size	-0.615	0.485	1.609	0.541 (0.209, 1.399)	0.205
Education level	-0.692	0.311	4.949	0.501 (0.272, 0.921)	0.026 *
Livelihood	0.205	0.304	0.453	1.228 (0.676, 2.227)	0.501
Distance	0.082	0.315	0.068	1.085 (0.585, 2.013)	0.794
Residency period	0.341	0.329	1.073	1.406 (0.738, 2.680)	0.300
Relationship	-0.103	0.330	0.098	0.902 (0.472, 1.723)	0.755
Conservation action					
Gender	0.600	0.245	6.001	1.822 (1.127, 2.945)	0.014 *
Age	-0.467	0.964	0.235	0.627 (0.095, 4.147)	0.628
Marital status	-0.298	0.254	1.380	0.742 (0.451, 1.221)	0.240
Household size	0.263	0.493	0.285	1.301 (0.495, 3.419)	0.593
Education level	-1.125	0.319	12.460	0.325 (0.174, 0.607)	< 0.001***
Livelihood	-0.797	0.308	6.704	0.451 (0.246, 0.824)	0.010*
Distance	-1.068	0.325	10.772	0.344 (0.182, 0.650)	0.001 **
Residency period	-0.079	0.332	0.057	0.924 (0.482, 1.771)	0.811
Relationship	0.593	0.337	3.097	1.809 (0.935, 3.503)	0.078

Significance: * < 0.05; ** < 0.01; ***p < 0.001

### Conservation action

Our results showed that most respondents favored countering conservation threats (median = 4; interquartile range = 3.8–4.3; Fig. 2). Specifically, item-by-item analysis showed that law enforcement (87.3%, *n* = 331), participation of the local community in conservation (81.3%, *n* = 308), conservation education and awareness (80.7%, *n* = 306), protected area establishment (80.2%, *n* = 304), ecotourism establishment (76.5%, *n* = 290), and avoiding hunting practices (63.6%, *n* = 241) were the top actions according to the respondents (Supplementary Table S2). Conservation action was negatively correlated with gender (*r* = -0.15; *p* < 0.01) and distance (*r* = -0.13; *p* < 0.05), while it was positively correlated with education level (*r* = 0.31; *p* < 0.001) and livelihood (*r* = 0.22; *p* < 0.001). Our data for logistic regression analysis also satisfied model adequacy (χ² = 9.99, df = 18, *p* > 0.05). Women respondents had significantly higher support for the proposed conservation action compared to men (β = 0.600, OR = 1.822, *p* < 0.05). Compared with literate, non-literate respondents negated the factors that contribute to conservation action (β = -1.125, OR = 0.325, *p* < 0.001). Respondents who depended entirely on farming and natural resource extraction were not in favor of the factors selected for conservation action (β = -0.79, OR = 0.451, *p* = 0.05). Similarly, the model showed that as the distance between respondents and the habitat of the species increased, their knowledge of and attitudes toward conserving the species declined (β = -1.068, *p* < 0.01) (Table 4).

### Cultural value of the species: medicinal and mythological insights

In this research, we also assessed hunting pressure as well as the cultural and mythological perspectives of participants through a thematic analysis. All interviewees (*n* = 25) reported that Harwood’s Francolin and related spurred galliform species, including chickens and roosters, are considered clean and acceptable within the context of cultural beliefs. This characteristic also helps communities in distinguishing adult males from females in Harwood’s Francolin (locally called “Soren” (ሶረን), “Sorene” (ሶረኔ) or “Sorit” (ሶሪት); all meaning ‘red’). Consequently, local people hunt these species while avoiding many other wild birds such as owls, buzzards, ravens, eagles, and vultures, despite their abundance in the area. According to the participants, this ethno-ornithological knowledge was associated with religious beliefs. Specifically, through Key Informant Interviews (KIIs), elderly clergymen and a farm healer shared their knowledge about the value of the species: its medicinal value was mentioned for asthma, fever, epilepsy, and hepatitis treatments.

With respect to mythological perceptions, the following quote from a participant highlights the key point:

*“*Soren is an exquisitely beautiful bird. Her splendor glows in the crimson skin around her eyes, in her vivid red legs and bill, and in the dark, lace-like patterning of the feathers that mantle her back, each delicately edged as though embroidered by nature’s own hand. Every hue upon her seems carefully brushed into place, as though she were adorned with kohl and shaped into elegance itself. Because of this radiant charm, in our community a striking, red-complexioned young woman is praised as ‘gorgeous like Soren.’ Likewise, a proud red bull or ox is honored with the name ‘Soren,’ a tribute to her vivid grace and unforgettable beauty.*”*

Because of their external appearance, some discussants portrayed the species as a figure of radiant and untamed beauty. They explained that its elegance is deeply embedded in Ethiopian cultural expression, especially in songs that celebrate love and devotion. Her presence reverberates most powerfully in the music of the legend Ejigayehu Shibabaw (GiGi) (Table 5) and Tewodros Kassahun (Teddy Afro) (Supplementary Table S3), where it stands as a symbol of romantic longing, emotional depth, and proud identity. The following GiGi’spoem captures the essence of love, longing, and identity, resonating with the themes explored in Ethiopian cultural expressions of devotion and romantic yearning.

**Table 5:** Expression of love in the context of Ethiopian culture in Meskel Flower (አደይ አበባ) song by Ejigayehu Shibabaw (GiGi)

Meskel Flower Sissy, a Meskel Flower, A feather of *Sorit*, Sissy, I thought you were a daisy bloom, I spent the day weeding Teff. Ho! Ho! Ho! Kiss me in time, my beloved, I winnow teff, yet I am enslaved. To love an enslaved soul—being bound— Is like a straw blown to burn. A handsome boy leads his herds to the hills; While I wither away, lost in the shadows of his neglect. Ho! Ho! Ho! June arrives with thunderstorms; You, cute boy, wear your *Gessa,* For with you, I long to share your flame of love. Owner of rifle; a good-looking boy, You are my fortune, my groomsman of love. Ho! Ho! Ho! A garland of fragrant grass, adorned with a Meskel Flower and *Sorit*; Saying “*Enkutatash*!”, I sent it to his home. My love, I yearn for the grove where we once spent the day, The flowers that bloomed there still emit their sweet scent. In my homestead, I saw a blossom of aromatic grass bloom, Armed with a Beljig sword and crowned with a shield. Ho! Ho! Ho! September, lush and green, September, lush and green, Radiant *Enkutatash*, bringing joy to the world. Joyous the festival of the True Cross and Saint John; Blessed is the day it is celebrated. Loving you, I shall remain; Hating you, I would fade away. (Translated from Ejigayehu Shibabaw (GiGi), 1998)	አደይ አበባ እቴ አደይ አበባ፣ የሶሪት ላባ። እቴ አደይ ነው ብዬ ፣ ጤፍ አርሞ ውዬ። አሆሆሆ… አንተ የከንፈር ወዳጅ በጊዜ ሳመኝ፣ ጤፍ አበጥራለሁ የሰው ግዙ ነኝ። የሰው ግዙ ሆኖ የሰው ግዙ መውደድ፣ እሳት በገለባ እፍ ብሎ መንደድ። ሸጋ ልጅ ወረዳ ወረዳ ደረባ፣ እኔን ሊያመነምን ከብቶቹን ሊያረባ። አሆሆሆ… ሰኔ አስገመገመ ጉብል ገሳ ልበስ፣ ከፍቅርህ ወላፈን አብሬህ ልቋደስ። ባለምንሽሩ ጎብላሌ ጎብላሌ፣ የፍቅሬ ባልደራስ አንተው ነህ እድሌ። አሆሆሆ… እንግጫ ጎንጉዬ ባደይ በሶሪት፣ እንቁጣጣሽ ብዬ ቤቱ ላኩለት። ሆዴ የዋልንበት ጫካው ናፈቀኝ፣ ዱሩ ያበቀለው አበባው ሸቶኝ። አየሁ ከደጃፌ እንግጫ ደንፍቶ፣ ቤልጂግ ጎራዴውን ጋሻጦሩን ደፍቶ። አሆሆሆ… መስከረም ለምለሙ፣ መስከረም ለምለሙ፣ ብሩህ እንቁጣጣሽ ደስታ ለአለሙ። እሰይ መስቀል ጠባ ቅዱስ ዮሐንስ፣ ብወድህ ኖራለው ብጠላህ መሰስ። እጅጋየሁ ሽባባው (ጂጂ), 1990 ዓም)

In addition, Umer Ali, a master of blending tradition and emotion, skillfully wove the rich melodies of Oromo and Amharic music into his heart-stirring song *“Zemuye”*, meaning *my sweetheart* (Table 6). Through this masterpiece, Umer intertwined the powerful symbols of the bird and the oxen, representing both freedom and hard work, to convey his deep, unspoken love. His fusion of melodies and symbolism brings to life a narrative of love that transcends language, much like the beautiful verses that follow.

**Table 6:** Folk poetic expression of romantic yearning in My Sweetheart (ዘሙየ!) song by Umer Ali

My Sweetheart! Oh, my love, leave it! Oh, sweetheart, the neck of a bird. You resemble *Soren*, your father’s ox, You resemble *Megal*, your father’s ox. Allowing others to say of me; He lost his mind in love. Not even a daughter of Wello; With a neck curved like a crystal goblet, One can be beaten for stealing a kettle. My love! My dear! Do not love your mother only for nine months; I have carried you unto eternity. From Ayteyef, Kemese is visible, What can a person do in Dessie, if he does not know love? (Translated from Umer Ali, 2016)	ዘሙየ! አረ ተይ ዘሙየ! አረ ዘሙ አንገት ወፍ! ያባትሽን በሬ ሶረንን መስለሽ፣ ያባትሽን በሬ መጋልን መስለሽ፣ ይኸ ልጅ አበደ ታስብይኝኛለሽ። እንኳን ለወሎ ልጅ ላንገተ ብርጭቆ፣ ሰው ይደበደባል ማንቆርቆርያ ሰርቆ። ዘሙየ! ዘምዋ! እናትሽን አትውደጅ ለዘጠኝ ወር እድሜ፣ እኔ አርግዤሻለሁ እስከዘልዓለሜ። አይጠየፍ ሆኖ ይታያል ከሚሴ፣ ፍቅር ያልገባው ሰው ምን ያደርጋል ደሴ። (ኡመር አሊ, 2009 ዓ/ም)

Among legendary singers and local communities, the very name of this species is spoken like poetry—an emblem of nature’s splendor. It is celebrated amid breathtaking landscapes, especially during the flowering season. At dawn and dusk, when the bird releases its tender breeding calls into the quiet air, its voice seems to bless the land itself, as though nature were singing its own love song. With its stunning presence and the serene landscapes it inhabits, it has captured the imagination, inspiring its use as a distinguished trademark in alcoholic beverages (Supplementary Figure S1) and as a beloved name for hotels.

## Discussion

### Status and value of Harwood’s Francolin

Our results shed new light on the socio-economic, demographic, spatiotemporal and behavioral contexts of ethno-ornithology in the Upper Blue Nile Basin (UBNB). This study revealed that the knowledge of respondents toward the status of Harwood’s Francolin was significantly predicted by socio-economic and demographic variables, in line with our hypothesis (H1). Specifically, unmarried respondents were better informed about the status of the species, possibly due to their dual roles in domestic and outdoor tasks that facilitate interactions in multidirectional socio-ecological contexts. Respondents living in proximity to the habitat of the species demonstrated greater knowledge of its status and value than those living farther away (H2). Interestingly, despite being a species with moderate sexual dimorphism, several non-literate respondents identified the sex of Harwood’s Francolin and other related pheasant species mainly by their spurs, color, size and vocalization, which are discriminant traits in the taxonomy, biogeography and behavioral ecology of birds. In addition, our regression models revealed that respondents with existing relationships demonstrated significantly greater knowledge of the status and value of the species than those without such experience (H3). Such socio-ecological patterns have been noted across different contexts and species [59], supporting the general role of interaction or prior experience in identifying ecological knowledge relevant to the conservation of threatened bird species [21].

The value of bird species is categorized into consumptive and non-consumptive values [6, 60, 61]. The knowledge and attitudes of respondents toward the value of Harwood’s Francolins comprised both instrumental (household consumption, market exchange and medicinal value) and non-instrumental (trademark, aesthetic, birdwatching, symbolic, role in classical music, and environmental indicator) values. The meat and eggs of pheasant species are a rich source of protein [9–60]. Accordingly, nearly all respondents (93.4%) stated that Harwood’s Francolin is hunted for household consumption. Hunting is common in late winter and spring, when the birds are in their breeding season and are often found in or near farmlands [43]. In Ethiopia, where people have a deep connection with nature, both plants and animals are also used for medicinal purposes [37–40], with plant-based remedies more commonly employed. Harwood’s Francolin is used for general internal ailments and is particularly valued as a treatment for asthma, fever, epilepsy and hepatitis. Informants reported that most francolin buyers used them for medicinal purposes in towns. Similarly, a congeneric species, Erckel’s Francolin also known as Erckel’s spurfowl (*Pternistis erckelii*) is believed to heal internal problems and associated diseases such as sexually transmitted infections in Ethiopia [40]. Related species, such as the Double-spurred Francolin (*Pternistis bicalcaratus bicalcaratus*) in Burkina Faso, are traded in Africa for traditional cultural beliefs and ritual practices [8].

In the Ethiopian cultural context, the informants stated that pheasant species are selectively hunted and considered as acceptable edible birds. This ethno-ornithological knowledge likely stems from religious prescriptions on lawful foods found in scriptural authority (e.g., Leviticus 11: 13–19), suggesting a long history of culturally guided bird-human relationships and the transmission of traditional ecological knowledge across generations. For example, birds of prey and some waterbirds, regardless of their abundance or size, are often perceived as ugly, harmful, or ritually unclean, and are frequently described as lacking spurs, a perception reported in other societies [18]. Nevertheless, in cases of illness or spiritual benefits for beliefs, people may consume any animal in the area [40], a practice that bypasses the moral norms associated with moralizing high gods in Ethiopia. Thus, religious norms are not isolated prescriptions but are integrated within broader socio-cultural and environmental frameworks [62].

Human–bird associations are often reflected in metaphors [63] and myths [61, 64], which relate to the birds’ broader range of traits [65]. Birds capture human attention through their vivid coloration, distinctive forms, captivating movements, melodious sounds, notable behaviors, and the cultural meanings people attach to them [4, 65–67]. As such, the colors and patterns of some birds are interpreted as symbols of vitality and elegance, while others are seen as signs of danger or bad luck [67]. Triangulated analyses showed that the socio-cultural significance of Harwood’s Francolin in Ethiopia is deeply rooted in its symbolic representation of beauty, trademark, love, and identity, largely associated with its graceful plumage. Its striking red facial skin, red legs, and bill glow brilliantly, while the dark to brown, lace-patterned feathers across its back add an air of refined elegance. In addition, we argue that the vernacular name and the endemic nature of our model species may shape human aesthetic preferences in the region, especially since ubiquitous, gray and black colored, and loud birds typically evoke negative perceptions [65, 67]. In general, pheasants are known for their elaborated plumage colors, which is also reflected in the English and scientific names of some African species, such as Handsome Francolin (*Pternistis nobilis*). In contrast, as in many other cultures, birds with entirely or uniformly black plumage, like ravens, are often viewed unfavorably and linked to harmful omens [66]. Therefore, our results also confirmed that Harwood’s Francolin is celebrated in Ethiopian music, where it symbolizes romantic longing and emotional depth. Its noticeable crepuscular activity and periods of vocalization often align with the Ethiopian temporarily green landscape during the wet season, reinforcing its symbolic connection to nature’s beauty and serenity, as reflected in the Ethiopian songs “Meskel Flower” and “So Stunning!” (Supplementary Table S2). This cultural fascination extends into the commercial sphere, where the bird is used as a trademark for alcoholic beverages (Supplementary Figure S1) and as a name for cattle and hotels, implying its broad cultural and economic influence.

Corroborating our results on the status and value of the species, previous studies show that human–bird interactions depend on species traits, especially those linked to aesthetic appeal and local significance, which are connected with positive perceptions [65]. Collectively, our findings indicated that Harwood’s Francolin occupies a multifaceted role in Ethiopian society, extending beyond consumption to encompass symbolic, medicinal, and ritual significance. This multifaceted role underscores a profound interconnection between ecological presence, cultural identity, and daily human livelihoods, demonstrating that the species is not only biologically important but also deeply rooted in socio-cultural systems. The charismatic nature of the species, coupled with its ecological and cultural prominence explain the strong local interest in its conservation and highlights the ways in which biodiversity and human culture are intimately intertwined.

### Direct and indirect human disturbances

This study provides the first systematic assessment of threats to the species by integrating traditional ecological knowledge from local communities. Accordingly, respondents reported hunting as the key conservation threat to the species. Likewise, egg collecting was regarded as another substantial threat, which is similar to other related species in Africa [68], Handsome Francolin; [69], Chestnut-naped Francolin *Pternistis castaneicollis atrifrons*] and Asia [24, Black Francolin *Francolinus francolinus francolinus*]. In our regression model, the main predictors of this knowledge were gender and residency period of respondents. Consistent with studies reporting gender-based differences in wildlife use, conservation and management [18, 70–72], our findings showed that men were significantly more likely to recognize hunting pressure as a major cause of species decline in the UBNB. Due to the community’s traditional division of labor, hunting is largely practiced by men, whereas women bear overall household responsibilities such as collecting firewood and fetching water. These culturally defined roles likely shape exposure to and knowledge of hunting activities, highlighting how local traditions influence perceptions of wildlife conservation threats in Ethiopia. As hypothesized, residency period significantly shaped the respondents’ awareness of direct human disturbances affecting the species (H4). This indicated that individuals who had lived longer in the area possessed greater awareness of such human disturbances affecting the species, likely due to their previous experience and this was linked to positive attitudes toward wildlife conservation. Similarly, other reports showed that long-term exposure to wildlife among communities living adjacent to protected areas can enhance a positive perception of wildlife [73].

According to respondents, the key drivers of change in habitat quality were vegetation clearance, fire, charcoaling, and farming. Previous ecological studies have shown that the decline of Harwood’s Francolin [25–27, 35] and other francolin species in the region [74] is linked to multiple anthropogenic disturbances. In our ethno-ornithological study, education level proved to be a significant predictor in the regression model. Specifically, the finding reflected that education facilitates access to socio-ecological information. This finding concurs with previous studies demonstrating that educated respondents had positive attitudes toward wildlife conservation [73, 75].

### Conservation action

The respondents largely supported the proposed conservation indicators, emphasizing the need to limit hunting, engage local communities, enforce laws against human encroachment, raise conservation awareness, establish protected areas, and promote ecotourism. Based on informants, some farming areas currently serve as the habitats for Harwood’s Francolins, with several individuals encountered, suggesting that the species has extripated in some areas. Although most women did not participate in hunting practices due to social norms and perceived risks in the wild, they showed greater knowledge of measures to reverse menacing factors for Harwood’s Francolin, in concordance with prior studies on other species elsewhere [70, 76–78]. Men were less supportive of the proposed measures due to their perceived restrictive nature of these measures, particularly with respect to “limiting hunting and selling” and “law enforcement.” These measures conflict with their routine activities linked to agriculture, livestock rearing, and the exploitation of wildlife resources, as is common in many other rural areas in Africa [77, 79]. Such closer involvement with natural habitats may reinforce domination-oriented attitudes among men [71], in which wildlife is valued primarily for its instrumental benefits rather than its intrinsic worth [70]. Interestingly, our model indicated that households primarily reliant on farming and natural resource extraction tended to exhibit negative attitudes toward species conservation. Local communities involved in business and formal employment were less dependent on resource extraction, including hunting, which may explain their comparatively more positive conservation attitudes, similar to previous studies [75].

In this study, literate individuals demonstrated greater knowledge of the species and its habitat conservation. This phenomenon is also observed in many wildlife studies [73]. Educational attainment corroborates regulatory mechanisms aimed at mitigating human impacts and fosters engagement in ecotourism initiatives that encourage local community participation in decision-making, underscoring the importance of education-centered conservation outreach. This is because alternative income for communities involved in conservation planning and ecotourism enhances positive attitudes toward wildlife conservation [75, 80]. Among communities living adjacent to wildlife habitats, species perceived as harmful or unattractive often attract negative attitudes and receive little protection [18, 81]. In contrast, species that provide benefits such as food, aesthetic attraction, economic value, symbolic significance, or ecosystem services, such as certain mammals [81], birds of prey [82], and pheasants [24], are more highly valued and more likely to be conserved. This supports our hypothesis that proximity to the habitat of the species is associated with greater awareness of its conservation status, suggesting that direct interaction can enhance local ecological knowledge and promote conservation actions. In general, birds used for food and cultural and religious beliefs play a key role in developing successful conservation strategies [8].

## Conclusions and recommendations

The local communities demonstrated a strong understanding of and positive attitudes toward the status and value of Harwood’s Francolins. They were also aware of the negative impacts of hunting and habitat destruction. The species holds strong biocultural charisma in Ethiopian society, with food, mythological, ritual, medicinal, folkloric and ecological, significance, and is often featured in romantic music. This socio-cultural prominence makes it an ideal flagship species for the conservation of other pheasants and the wider ecosystems of the Upper Blue Nile Basin. Local communities support its protection through restriction on hunting, law enforcement, community initiatives, and protected areas, which also promote livelihoods and ecotourism. Therefore, conservation planning should include fostering education and awareness among local communities, mainly by restricting hunting and egg collecting during the breeding season and we also urge gender-inclusive conservation strategies in the area. We also urge further study about the medicinal and mythological value of pheasant species in Ethiopia.

## Supplementary Information

Supplementary Material 1

## Data Availability

The datasets used and/or analyzed during the current study are available from the corresponding author on reasonable request.
