# Supplementary material for: Hidden charisma: ethno-ornithological knowledge and attitudes of local communities toward a threatened endemic pheasant in the Upper Blue Nile Basin, Ethiopia
Source: J Ethnobiol Ethnomed. 2026 Sep 17;22:88. doi: 10.1186/s13002-026-00942-0 (PMC13587477; doi:10.1186/s13002-026-00942-0)
Supplement: Supplementary file 1 [file 13002_2026_942_MOESM1_ESM.doc]

Hidden charisma: Ethno-ornithological knowledge and attitudes of local communities toward a threatened endemic pheasant in the Upper Blue Nile Basin, Ethiopia

Abadi Mehari Abrha^1,2,3^, Kai Gedeon^2^, Lars Podsiadlowski^2^; Temesgen Tigabu Derso^4^, Tesfaye Mesele^5^, Till Töpfer^2*^

^1^Institute for Evolutionary Biology and Ecology, University of Bonn, An der Immenburg 1, 53121 Bonn, Germany

^2^Leibniz Institute for the Analysis of Biodiversity Change, Museum Koenig Bonn, Adenauerallee 127, 53113 Bonn, Germany

^3^Department of Biodiversity and Ecotourism Management, Mekelle University, P.O. Box 231, Mekelle, Tigray, Ethiopia

^4^Ethiopian Biodiversity Institute, P.O. Box 30726, Addis Ababa, Ethiopia.

^5^Department of foreign languages and literature, Mekelle University, P.O. Box 231, Mekelle, Tigray, Ethiopia

* Corresponding author. E-mail address: T.Toepfer@leibniz-lib.de (Till Töpfer)

Table S 1. Household population size (N), sample size (n), margin error (%) and final collected and accepted sample size.

| District | Population size (N) | Sample size (n) | Margin error (%) | Final sample size (complete) |
| --- | --- | --- | --- | --- |
| Merahbete | 906 | 90 | 9.8% | 87 |
| Moretna Jiru | 228 | 36 | 15.0% | 34 |
| Basona Worena | 466 | 40 | 14.8% | 40 |
| Gosamin | 580 | 63 | 11.7% | 58 |
| Debre Elias | 344 | 40 | 14.6% | 37 |
| Kelela | 411 | 68 | 10.9% | 62 |
| Sayint | 454 | 63 | 11.5% | 61 |
| Total | 3,389 | 400 | 4.6% | 379 |

Table S2. Category of response variables (status, values, hunting pressure, habitat destruction and conservation action) and their Cronbach’s alpha coefficient α values. Item frequency (percentages) were calculated from the respondents’ responses (n = 379).

| Category  and  acronym | Item | Cronbach's Alpha | Yes | No | Unsure |
| --- | --- | --- | --- | --- | --- |
| 1. Status of the species (α = 0.93) | | | | | |
| HF | Harwood’s Francolin exists in your locality. | 0.91 | 76.5% (n = 290) | 4% (n = 15) | 19.5% (n = 74) |
| SEE | The species is an endemic to Ethiopia. | 0.88 | 70.4% (n = 267) | 1.1% (n = 4) | 28.5% (n = 108) |
| DEC | The species is currently declining in its native habitat. | 0.89 | 65.2% (n = 247) | 7.6% (n = 29) | 27.2% (n = 103) |
| 2. Values of the species (Instrumental and non-instrumental values) (α= 0.82) | | | Disagree | Neutral | Agree |
| HC | The species is hunted for household consumption. | 0.82 | 0.5% (n = 2) | 6.1% (n = 23) | 93.4% (n = 354) |
| MEX | Local people hunt the species for market exchanges. | 0.78 | 6.6% (n = 25) | 33.5% (n = 127) | 59.9% (n = 227) |
| MV | The species is hunted for medicinal value. | 0.78 | 11.3% (n = 43) | 38.0% (n = 144) | 50.7% (n = 192) |
| BW | Bird watchers are visiting the species in the area. | 0.81 | 28.2% (n = 107) | 39.8% (n = 151) | 31.9% (n = 121) |
| STV | The species is important for trademark, aesthetic, and symbolic values. | 0.77 | 10.6% (n = 40) | 25.1% (n = 95) | 64.4% (n = 244) |
| CMC | The bird has played a role in classical music in the country. | 0.77 | 7.7% (n = 29) | 44.1% (n = 167) | 48.3% (n = 183) |
| IE | The species is important as environmental indicator | 0.80 | 19.3% (n = 73) | 50.7% (n = 192) | 30.1% (n = 114) |
| 3. Hunting pressure (α= 0.81) | | | | | |
| HUDS | Hunting decreases the species in the area. | NA | 10.6% (n = 40) | 25.1% (n = 95) | 64.4% (n = 244) |
| EC | Egg collecting decreases the species in the area. | NA | 13.2% (n = 50) | 39.8% (n = 151) | 47.0% (n = 178) |
| 4. Habitat destruction (α= 0.74) | | | | | |
| CUTFIRE | Cutting trees for firewood preparation is well known in the habitat. | 0.72 | 3.7% (n = 14) | 9.8% (n = 37) | 86.5% (n = 328) |
| VEGS | There is vegetation clearance for settlement. | 0.74 | 35.4% (n = 134) | 28.5% (n = 108) | 36.1% (n = 137) |
| VEGFH | There is vegetation clearance for fence and house. | 0.72 | 16.1% (n = 61) | 22.2% (n = 84) | 61.7% (n = 234) |
| CHARPRA | Charcoal preparation is practiced for different purposes. | 0.73 | 12.9% (n = 49) | 30.3% (n = 115) | 56.7% (n = 215) |
| FIRE | The habitat of the species is threatened by introduction of fire. | 0.72 | 26.6% (n = 101) | 44.3% (n = 168) | 29.0% (n = 110) |
| FARM | The habitat of the species is disturbed by farming activities. | 0.72 | 14.0% (n = 53) | 16.4% (n = 62) | 69.7% (n = 264) |
| CC | Climate change (drought) affects the habitat of the species. | 0.72 | 34.6% (n = 131) | 46.7% (n = 177) | 18.7% (n = 71) |
| LG | Livestock grazing affects the habitat of the species. | 0.71 | 14.5% (n = 55) | 36.7% (n = 139) | 48.8% (n = 185) |
| SD | Soil denudation affects the potential habitat of the species. | 0.71 | 49.1% (n = 186) | 36.4% (n = 138) | 14.5% (n = 55) |
| MP | Mining practices dwindle the habitat. | 0.72 | 43.5% (n = 165) | 42.0% (n = 159) | 14.5% (n = 55) |
| TM | Thatching and mowing poses pressure on the habitat the species. | 0.71 | 22.2% (n = 84) | 51.5% (n = 195) | 26.4% (n = 100) |
| 5. Conservation action (α = 0.79) | | | | | |
| HUME | Hunting and selling the species should be limited. | 0.75 | 6.9% (n = 26) | 29.6% (n = 112) | 63.6% (n = 241) |
| CES | Local community engagement in community-based conservation areas can help species-habitat conservation. | 0.75 | 0.8% (n = 3) | 17.9% (n = 68) | 81.3% (n = 308) |
| LAW | Effective and efficient law enforcement regulations can assist to curb human encroachment due to farming, population growth, and settlement in the area. | 0.76 | 2.4% (n = 9) | 10.3% (n = 39) | 87.3% (n = 331) |
| EDU | Conservation education awareness about the species and habitat is crucial. | 0.76 | 0.5% (n = 2) | 18.7% (n = 71) | 80.7% (n = 306) |
| PA | Establishing of protected area may help the existence of the species. | 0.76 | 2.4% (n = 9) | 17.4% (n = 66) | 80.2% (n = 304) |
| ECO | Ecotourism expansion in the area can help species and habitat conservation. | 0.76 | 0.5% (n = 2) | 23.0% (n = 87) | 76.5% (n = 290) |

Table S3. Expression of love in the context of Ethiopian culture in So Stunning! (ያምራል!) song by Tewodros Kassahun (Teddy Afro).

| So Stunning!  No space could contain it when your beauty stood out,  Shining like the sun.  Painting brows around your eyes,  Like a stroke of kohl.  How marvelous you are, ruby-like,  May your beauty shine for all the world to see.  When I behold the rose upon your lips,  I lose myself completely.  Left stunned, with nothing left to do,  My eyes were drawn to your mouth.  "Kiss her, kiss her," it urged me,  Yet I mastered myself once again.  Lips that called, "Kiss me, kiss me,"  Suddenly made me forget myself.  Though my heart refused to listen when beauty called out,  Saying to me, "Come, come and see me..."  How could I have remained apart from myself,  When it called to me, "Come, come and see me..."  It enchants the heart and overwhelms the soul,  Your beauty is astonishingly radiant.  So stunning, so stunning,  Your elegance shines.  So stunning, so stunning,  Your beauty is painfully beautiful.  My heart lost its heart,  My senses slipped away,  When your beauty called to me,  Saying to me, "Come, come and see me."  The beauty of the forest grove,  The feather of *Sorit*,  Who made you bloom in Addis Ababa?  Amazed and dazed, I stood without words,  My gaze was drawn to your mouth.  "Kiss her, kiss her," it urged me,  Yet I mastered myself once again.  Lips that called, "Kiss me, kiss me,"  Suddenly made me forget myself.  Though my heart refused to listen when beauty called out,  Saying to me, "Come, come and see me..."  How could I have remained apart from myself,  When it called to me, "Come, come and see me..."  It enchants the heart and overwhelms the soul,  Your beauty is astonishingly radiant.  So stunning, so stunning,  Your elegance shines.  So stunning, so stunning,  Your beauty is painfully beautiful.  My heart lost its heart,  My senses slipped away,  When your beauty called to me,  Saying to me, "Come, come and see me."  (Tewodros Kassahun (Teddy Afro), 2017) | ያምራል!  ሦፍራ ጠበበው ያንቺ ውበት ጎልቶ ሲታይ፣  ፈክቶ እንደ ፀሀይ።  ውብ ያደረገሽ ባይንሽ ዙሪያ ቅንድብ ስሎ፣  ኩል አስመስሎ።  እንዴት ታምሪያለሽ ሩቢ መሳይ፣  ውበትሽ ወጥቶ ለአለም ይታይ፣  ከከንፈርሽ ላይ ፅጌሬዳ ሳይ ፣  ከራሴ ጋራ እስከምለያይ ።  በብቀር ፈዝዤ ጠፍቶኝ ማደርገው ፣  አይኔን ከአፍሽ ላይ ጣል ባረገው ።  ሳማት ሳማት አለኝ እና ፣  ቀልቤን ገዛሁ እንደገና ።  ሳሙኝ ሳሙኝ የሚል ከንፈር ፣  ድንገት አጉል አርጎኝ ነበር ።  አልሰማ ቢል ልቤ ውበት ሲጣራ ፤ ሲለኝ ናና እየኝ  መች እለያይ ነበር ከራሴ ጋራ ፤ ሲለኝ ናና እየኝ  ልብ አሸፍቶ ቀልብ ያሰውራል፣  ቁንጅናሽ ለጉድ ያምራል ።  ያምራል ያምራል  ቁንጅናሽ ያምራል፣  ያምራል ያምራል  ውበትሽ ያማል ።  ልቤ ልብ አጣ  ቀልቤን ሰወረኝ ፣  መልክሽ ሲጣራ  ሲለኝ ናና እየኝ ።  የደን አጸድ ጌጥ የሶሪት ላባ፣  ማን አበቀለሽ ካዲስ አበባ?  ገርሞኝ ፈዝዤ ጠፍቶኝ ማደርገው ፣  አይኔን ከአፍሽ ላይ ጣል ባረገው ።  ሳማት ሳማት አለኝ እና ፣  ቀልቤን ገዛሁ እንደገና ።  ሳሙኝ ሳሙኝ የሚል ከንፈር ፣  ድንገት አጉል አርጎኝ ነበር ።  አልሰማ ቢል ልቤ ውበት ሲጣራ ፤ ሲለኝ ናና እየኝ  መች እለያይ ነበር ከራሴ ጋራ ፤ ሲለኝ ናና እየኝ  ልብ አሸፍቶ ቀልብ ያሰውራል ፣  ቁንጅናሽ ለጉድ ያምራል።  ያምራል ያምራል፣  ቁንጅናሽ ያምራል  ያምራል ያምራል  ውበትሽ ያማል ።  ልቤ ልብ አጣ  ቀልቤን ሰወረኝ ፣  መልክሽ ሲጣራ  ሲለኝ ናና እየኝ ።  (ቴዎድሮስ ካሳሁን (ቴዲ አፍሮ), 2009 ዓም) |
| --- | --- |

Figure S1. Trademark for alcoholic beverage locally known as Areki.
